# Draft Genome Sequence of *Lewinella* sp. Strain 4G2 Isolated from the Coastal Sea Surface Microlayer

**DOI:** 10.1128/genomeA.00754-16

**Published:** 2016-07-28

**Authors:** Shu-Kuan Wong, Susumu Yoshizawa, Yu Nakajima, Yoshitoshi Ogura, Tetsuya Hayashi, Koji Hamasaki

**Affiliations:** a Division of Marine Life Science, Department of Marine Ecosystem Dynamics, Atmosphere and Ocean Research Institute, The University of Tokyo, Kashiwa, Chiba, Japan; b Department of Bacteriology, Faculty of Medical Sciences, Kyushu University, Fukuoka-shi, Fukuoka, Japan

## Abstract

We report here the draft genome of *Lewinella* sp. strain 4G2, isolated from the sea surface microlayer (SML) of a coastal marine inlet. The genome sequence of strain 4G2 should contribute to understanding the lifestyles of bacteria living in the SML.

## GENOME ANNOUNCEMENT

The sea surface microlayer (SML), defined as the topmost 1,000 µm of the sea surface, has been found to possess different physicochemical and biological properties from the underlying water directly below it (1). To date, the microbiology of the SML still remains poorly understood, with only one representative SML isolate having been characterized so far (2). Here, we present the draft genome sequence of a novel strain, designated 4G2, isolated from the SML of a coastal marine inlet.

Strain 4G2 was isolated from the SML of Aburatsubo Inlet, Misaki, Japan. Water samples from the SML (42-µm depth) were collected using the glass plate method (3) and isolated using methods described previously (4). Genomic DNA was extracted using phenol-chloroform and ethanol precipitation (5). An 800-bp paired-end library and an 8-kb mate-pair library were prepared using the Nextera XT DNA library preparation kit (Illumina) and Nextera mate-pair sample preparation kit (Illumina), respectively; 300 bp of each end of the libraries were sequenced on the MiSeq instrument with the MiSeq Reagent kit version 3 (Illumina). A total of 870,144 mate-paired and 1,793,514 paired-end reads were assembled using Platanus version 1.2.4 (6). The sequences were annotated automatically using the NCBI PGAAP (7) and reviewed with RAST version 2.0 (http://rast.nmpdr.org).

The 16S rRNA gene sequence taxonomic identification using EzTaxon (8) revealed that strain 4G2 belongs to the genus *Lewinella* and is most similar to *Lewinella antarctica* IMCC3223^T^ (95.72%) from the family *Saprospiraceae* in the class *Sphingobacteriia*. The *Lewinella* sp. strain 4G2 genome sequence consisted of 21 scaffolds (5,219,850 bp in total, *N*_50_ = 4,178,312 bp) with a median read coverage of 53.0×. The estimated genome size (5.22 Mb) of strain 4G2 was smaller than two other draft genome sequences of *Lewinella* strains available in GenBank (*L. persica* DSM 23188, 6.67 Mb, G+C 53.0%; *L. cohaerens* DSM 23179, 7.30 Mb, G+C 45.0%) (9) but with a higher G+C content (55.3%).

PGAAP identified 3,830 genes, including 3,776 protein-coding sequences (CDSs), 42 tRNAs, three noncoding RNA genes, and 28 pseudogenes. The RAST annotation also identified 3,768 CDSs. Strain 4G2 possessed 61 gene homologs responsible for DNA repair and 81 stress-response gene homologs, which are mostly involved in regulating heat shock and osmotic and oxidative stress and possibly play roles in helping the organism to cope with high radiation and temperature stresses in the SML. Interestingly, gene homologs involved in nitrate and nitrite ammonification as well as organic sulfur assimilation were present in the genome of strain 4G2 but not in the genomes of *L. cohaerens* and *L. persica*.

The draft genome sequence of *Lewinella* sp. strain 4G2 will provide further insight into the role of the organism in the SML, as well as the physiology and mechanism that the organism employs to cope with the living conditions in the SML.

### Nucleotide sequence accession numbers.

This whole-genome shotgun project has been deposited in DDBJ/EMBL/GenBank under the accession number LVWJ00000000. The version described in this paper is the second version, LVWJ02000000.
